# Speciation patterns of *Aedes* mosquitoes in the Scutellaris Group: a mitochondrial perspective

**DOI:** 10.1038/s41598-024-61573-7

**Published:** 2024-05-13

**Authors:** Antsa Rakotonirina, Catherine Dauga, Morgane Pol, Mallorie Hide, Linavin Vuth, Valentine Ballan, Sosiasi Kilama, Sylvie Russet, Sébastien Marcombe, Sébastien Boyer, Nicolas Pocquet

**Affiliations:** 1 Medical and Veterinary Entomology Unit, Institut Pasteur du Cambodge, Phnom Penh, Cambodia; 2 Unité de Recherche et d’Expertise en Entomologie Médicale, Institut Pasteur de Nouvelle-Calédonie, Nouméa, Nouvelle-Calédonie; 3 Arboriruses and Insect Vectors Laboratory, Institut Pasteur Paris, Paris, France; 4 Maladies Infectieuses et Vecteurs: écologie, génétique, évolution et contrôle (MIVEGEC), Université de Montpellier, IRD, CNRS, Montpellier, France; 5 Vector Borne Disease Laboratory, Institut Pasteur du Laos, Vientiane, Laos; 6 Vector Control Consulting-South East Asia SOLE CO., LTD., Vientiane, Lao PDR; 7 Ecology and Emergence of Arthropod-Borne Pathogens Unit, Department of Global Health, Institut Pasteur, CNRS UMR2000, Paris, France

**Keywords:** Scutellaris subgroup, Albopictus subgroup, Paleogenic period, *Cox*1 gene, Evolution, Genetics

## Abstract

The Scutellaris Group of *Aedes* comprises 47 mosquito species, including *Aedes albopictus*. While *Ae. albopictus* is widely distributed, the other species are mostly found in the Asia–Pacific region. Evolutionary history researches of *Aedes* species within the Scutellaris Group have mainly focused on *Ae. albopictus*, a species that raises significant public health concerns, neglecting the other species. In this study, we aimed to assess genetic diversity and estimate speciation times of several species within the Scutellaris Group. Mosquitoes were therefore collected from various Asia–Pacific countries. Their mitochondrial cytochrome c oxidase subunit 1 (*cox*1) and subunit 3 (*cox*3) sequences were analyzed alongside those of other Scutellaris Group species available in the GenBank database. To estimate the divergence time, we analyzed 1849 *cox*1 gene sequences from 21 species, using three species (*Aedes aegypti, Aedes notoscriptus* and *Aedes vigilax*) as outgroups. We found that most of the speciation dates occurred during the Paleogene and the Neogene periods. A separation between the Scutellaris Subgroup and the Albopictus Subgroup occurred approximately 64–61 million years ago (MYA). We also identified a split between species found in Asia/Micronesia and those collected in Melanesia/Polynesia approximately 36–35 MYA. Our findings suggest that the speciation of *Aedes* species within the Scutellaris Group may be driven by diversity in mammalian hosts, climate and environmental changes, and geological dynamics rather than human migration.

## Introduction

Within the Culicidae family, mosquitoes comprise over 3600 species, categorized into two subfamilies and 113 genera^1,2^. Among these genera, the *Aedes* genus, specifically the *Stegomyia* subgenus, hosts numerous vector species^3–6^. As an example, *Aedes* (*Stegomyia*) *aegypti,* belonging to the Aegypti Group, is a major vector of dengue virus (DENV) and can also transmit several other arboviruses, including chikungunya (CHIKV), Yellow fever (YFV) and Zika viruses (ZIKV)^5^. Another significant species within the *Stegomyia* subgenus is the invasive Asian tiger mosquito, *Ae.* (*Stegomyia*) *albopictus*. This species can transmit these four arboviruses and has also been associated with the transmission of more than 30 other arboviruses^6–8^.

*Aedes albopictus* belongs to the Scutellaris Group, which is subdivided in two Subgroups: Albopictus and Scutellaris. This group currently comprises a total of 46 species^2^ with at least 12 confirmed or potential vectors of arboviruses. For example, *Ae. *(*Stegomyia*) *malayensis,* found in Southeast Asia^9^, is a vector of DENV^10^ as well as *Ae.* (*Stegomyia*) *scutellaris*^11^*,* which is present in Southeast Asia and in the Pacific region^9,12^. *Aedes* (*Stegomyia*) *polynesiensis*, present in the eastern Pacific region^12^, can transmit CHIKV and ZIKV^3,4^ and is suspected to transmit DENV^13^. *Aedes* (*Stegomyia*) *hensilli,* an endemic species of Yap island, has the potential to transmit CHIKV and ZIKV^14,15^. Several other species exclusively found in the Pacific islands, including *Ae.* (*Stegomyia*) *cooki, Ae.* (*Stegomyia*) *hebrideus, Ae.* (*Stegomyia*) *kesseli, Ae.* (*Stegomyia*) *marshallensis, Ae.* (*Stegomyia*) *pseudoscutellaris, Ae.* (*Stegomyia*) *rotumae, and Ae.* (*Stegomyia*) *tabu*, are also suspected to be potential vectors of DENV^16^.

Understanding mosquito phylogeny holds significant importance for different reasons. It can significantly contribute to define their taxonomy and classification. The classification within the Scutellaris Group species remains a topic of debate, due to their morphological resemblances and potential hybridization of some species observed in laboratory settings without a decrease in fertility^17,18^. Consequently, exploring their relationships through molecular investigations can be a crucial tool in solving these taxonomical issues. Dating phylogeny could allow a better understanding of the evolutionary history of these species, helping to identify the period of their speciation events.

Few studies have investigated the phylogenetic relationship among the species within the Scutellaris Group, primarily emphasizing slight genetic distinctions between some species even with the use of ribosomal (the internal transcribed spacer 2, the 16S and 28S ribosomal sequences) or mitochondrial (the cytochrome c oxidase subunit 1) genes^19–21^.

The understanding of the Scutellaris Group evolution is also limited due to the limited number of species used in previous investigations. When using mitochondrial data, these works have particularly emphasized the divergence among *Ae. polynesiensis* and *Ae. riversi* and between *Ae. albopictus, Ae. flavopictus* and *Ae. subalbopictus* during the Paleogene period (66–23 million year ago), along with the divergence between *Ae. dybasi* and *Ae. palauensis* during the Neogene period (23–2.58 million years ago)^22,23^.

In contrast to previous studies, our current research includes broader range of species within the Scutellaris Group from Asia and Pacific regions. Utilizing new sequence data, we assessed the genetic diversity among species of the Scutellaris Group. Furthermore, our investigation aims to estimate speciation time of these species, to better understand their evolutionary history.

## Methods

### Biological material

Adults and larvae mosquitoes (n = 42) belonging to the Scutellaris Group, were collected in five countries: Cambodia, Fiji, Laos, New Caledonia and Wallis & Futuna (Table 1). Field collections took place between March 2016 and June 2022. Various sampling methods, including the BG-Sentinel^®^ trap, BG-Gravid^®^ *Aedes* trap (Biogents, Germany), and CDC light trap with CO_2_ (BioQuip, USA), were employed for adult collection. Larvae were collected directly from their breeding sites using water pipettes and plastic cups and reared in the laboratory until adults at 28 °C ± 2 °C and a relative humidity of 80 ± 10%. They were fed with Innovafeed ad libitum throughout this rearing period. Upon emergence, adults were collected using mouth aspirator and killed by freezing at – 80 °C. Morphological identification of all collected mosquitoes was performed under a stereo microscope using dichotomous identification keys^12,17,24^, followed by preservation at − 80 °C for subsequent analyses. Among these specimens, *Ae. unalom*, a newly discovered species within the Albopictus Subgroup, was included^25^. This species is morphologically close to *Ae. albopictus* but is genetically distinct. We also included *Ae. scutellaris*, a species that was recently introduced in New Caledonia^26^.

**Table 1:** Mosquitoes collected from each country.

Countries	Species	No. of specimens included in the analyses^a^	Date of collection
Cambodia	*Ae. albopictus*	5	February 2020
	*Ae. unalom*	5	June 2022
Fiji	*Ae. albopictus*	6	May 2018
	*Ae. pseudoscutellaris*	5	May 2018
Laos	*Ae. albopictus*	5	November 2019
	*Ae. malayensis*	2	May 2019
New Caledonia	*Ae. scutellaris*	4	March 2016
Wallis and Futuna	*Ae. futunae*	5	December 2020
	*Ae. polynesiensis*	5	November–December 2020

^a^Only 42 specimens were included in this study. The other specimens were used in another work.

### DNA extraction, PCR and sequencing procedures

Following the method outlined by Rakotonirina et al.^21^, DNA was individually extracted from each mosquito's body using a DNA Blood and Tissue Kit (Qiagen, Hilden, Germany) in accordance with the manufacturer's guidelines. The extracted DNA underwent amplification via polymerase chain reaction (PCR) utilizing specific primers targeting mitochondrial genes which are widely used markers in evolutionary and taxonomic studies due to the lack of DNA recombination, simplifying inheritance patterns, in contrary to the nuclear gene^27^. We focused on cytochrome c oxidase subunits 1, 2, and 3 (*cox*1, *cox*2, and *cox*3) and cytochrome b, all known to be under strong purifying selection in mosquitoes^27,28^. However, during our study, technical limitations resulted in interpretable data only for *cox*1 and *cox*3. Consequently, we present our findings based on these two genes.

Cytochrome c oxidase subunit 1 gene is 1537 basepairs (bp). For technical reasons, three sets of *cox*1 primers were used during this study. The first set of primers (LCO1490: 5′-GGT CAA CAA ATC ATA AAG ATA TTG G-3′ and HC02198: 5′-TAA ACT TCA GGG TGA CCA AAA AAT CA-3′)^21^ allow to amplify the 709 bp in the 5′ part of *cox*1, ranging from nucleotide 17 to nucleotide 725. The second *cox*1 primer set allowed amplifying 680 bp in the 3′ part of *cox*1, starting from nucleotide 688 to nucleotide 1367, with COS2183N (5′-CAR CAY YTA TTY TGR TTY TTY GG-3′) and COA3107N (5′-TCY ATT AAA GGA GAA GYW CTA TYT TG-3′) primers^20^. The last *cox*1 primer set starting at nucleotide 220 and ending at nucleotide 1508, generated a 1288 bp fragment using C1-J-1718 (5′-GGA GGA TTT GGA AAT TGA TTA GTT CC-3′) and C1R3 (5′-TAA GTA TGT TCT GCA GGA GG-3′) primers^25,29^. Moreover, *cox*3 gene is of 789 bp. The primers designed for this study (COIII-F: 5′-ACA GGG GCT ATT GGA GCT AT-3′ and COIII-R: 5′-GCT GCA GCT TCA AAT CCA AAA T-3′) enabled the amplification of 662 bp fragment, initiating from nucleotide 58 and extending to nucleotide 719.

PCR amplifications were conducted in 25 μl reactions, employing isolated DNA at concentrations between 10 ng/μl and 100 ng/μl as the template. Each PCR reaction comprised 0.2 μM of both forward and reverse primers, 1 × of HotStarTaq^®^ DNA Polymerase (Qiagen), and distilled water. The thermal cycling protocol involved several steps: an initial denaturation at 94 °C for 3 min, followed by 35 cycles. During each cycle, there was denaturation at 94 °C for 1 min and annealing at specific temperatures: 50 °C for 1 min (for LCO1490/HC02198), 52 °C for 1 min (for COS2183N/COA3107N), 51 °C for 40 s (for C1-J-1718/C1R3), and 61 °C for 1 min (for COIII-F/COIII-R). After annealing, extension step per cycle occurred at 72 °C for 1 min (for LCO1490/HC02198, COS2183N/COA3107N, and COIII-F/COIII-R), while for C1-J-1718/C1R3, extension was at 72 °C for 1 min 30 s. Finally, a concluding extension step was performed at 72 °C for 10 min. Verification of double-stranded PCR products was done through 1.5% agarose gel electrophoresis, and subsequently, PCR products underwent sequencing using the Sanger technique (conducted by Genoscreen, France, or Macrogen, Korea).

### Data analyses

The sequences obtained were initially assembled using PREGAP and GAP software (version 4.10.2)^30^. Following sequence quality control using the Bioedit software (version 7.2.5)^31^, the sequences were aligned, and the number of polymorphic sites for each gene was determined using the DNAsp (version 6.12) program^32^. A haplotype network for the concatenated gene was constructed using the Templeton Crandall and Sing (TCS) algorithm^33^ within popART (version 1.7)^34^.

The divergence dating analysis relied on a Bayesian Markov Chain Monte Carlo (MCMC) approach, conducted via BEAST (2.6.0 package)^35^. This aimed to infer the Scutellaris Group's topology and estimate the speciation time of the common ancestor of clades in millions of years (MYA). To achieve this, we used the *cox*1 gene due to its high representation in the GenBank database for mosquito species. Available *cox*1 sequences from various species of the Scutellaris Group (*Ae. albopictus, Ae. daitensis, Ae. downsi, Ae. dybasi, Ae. flavopictus, Ae. futunae, Ae. galloisi, Ae. hensilli, Ae. malayensis, Ae. miyarai, Ae. palauensis, Ae. polynesiensis, Ae. pseudoscutellaris, Ae. riversi, Ae. scutellaris* and *Ae. unalom*) originating from Asia and the Pacific region were retrieved from the GenBank database and were added to our newly sequenced data. We have removed sequences containing extensive ambiguous “N” symbols based on alignment analysis using the Bioedit software (version 7.2.5)^31^. A total of 1849 *cox*1 sequences were included in the analysis (Supplementary Table 1). *Aedes aegypti, Aedes notoscriptus* and *Ae. vigilax* were used as outgroups. The full list of *cox*1 sequences used in this study is available as Excel file (Supplementary Table 1). Two calibration points, representing the speciation event between *Ae. polynesiensis* and *Ae. riversi,* and between *Ae. notoscriptus* and *Ae. vigilax* were adopted based on previous research within the Culicinae subfamily, using mitochondrial full genome data^22^.

The best evolutionary model was selected by the ModelFinder^36^, followed by the tree reconstruction using the IQ-TREE version 1.6.12^37^ performing the ultrafast bootstrapping with 1,000 replicates. The Bayesian analysis comprised runs of 10 million generations, with tree sampling at every 1000 iterations for each alignment dataset. It used a Birth–Death process of speciation as the Tree Prior. Furthermore, the GTR + G + I evolutionary model was employed. The analysis of these two parts of the *cox*1 gene was conducted separately because the GenBank database only had sequences for most species in either the 5' or 3′ position. After discarding the initial 25% burn-in, the tree was obtained through the TreeAnnotator program^35^. For each speciation time, we calculated the highest posterior density (HPD), which represents a set containing 95% of the sampled values (95% HPD). Finally, the consensus trees were visualized and edited using FigTree (version 1.4.4)^38^.

## Results

### Sequence variation among the *Aedes* of Scutellaris Group

All the sequences newly generated during our study (40 *cox*1 and 42 *cox*3 sequences) were deposited in the GenBank database (accession numbers PP355451 to PP355490 and PP357893 to PP357934). This study provided the first *cox*3 sequences of six *Aedes* species of the Scutellaris Group: *Ae. futunae, Ae. malayensis, Ae. polynesiensis, Ae. pseudoscutellaris, Ae. scutellaris* and *Ae. unalom*.

After aligning the sequences and trimming the ends to ensure uniform sequence length across all individuals, the lengths of both the *cox*1 and *cox*3 regions decreased. Within the 5′ part of *cox*1 sequence (420 bp), a total of 102 nucleotide substitutions and 87 polymorphic sites were identified. Few nucleotide mutations were observed among different *Ae. albopictus* populations from Asia and Pacific countries (two–three mutations), as well as between *Ae. polynesiensis* from Wallis & Futuna and *Ae. pseudoscutellaris* from Fiji (five mutations), and between *Ae. malayensis* from Laos and *Ae. scutellaris* detected in New Caledonia in 2016 (two mutations) (Supplementary Table 2).

The 3′ part of *cox*1 gene (493 bp) presented 90 nucleotide substitutions and 79 polymorphic sites. Similar to the 5′ part of *cox*1 sequence, few nucleotide mutations were identified across the different *Ae. albopictus* populations (two mutations), between *Ae. polynesiensis* and *Ae. pseudoscutellaris* (two mutations), and between *Ae. malayensis* and *Ae. scutellaris* (five mutations) (Supplementary Table 3).

Within the *cox*3 gene sequence (553 bp), 110 mutations occurred with 101 polymorphic sites. Only a few mutations (three–four mutations) were detected among various *Ae. albopictus* populations. Only three mutations were identified between *Ae. polynesiensis* and *Ae. pseudoscutellaris* (Supplementary Table 4)*.*

**Figure 1:**
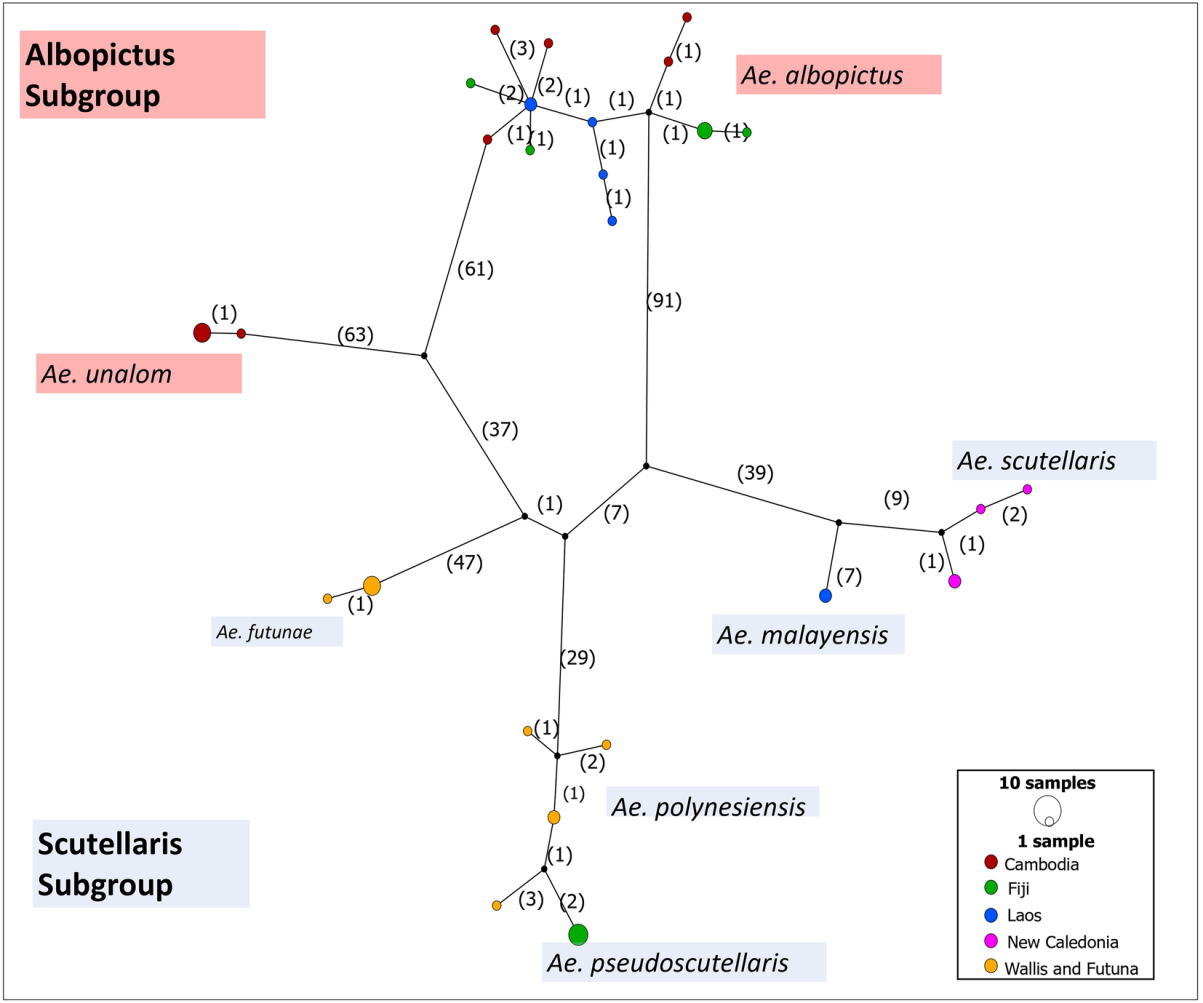
Haplotype network of species of Scutellaris Group (n = 42) using the concatenated *cox*1 and *cox*3 genes (1466 bp). Nodes in the graph represent distinct haplotypes, and their size indicate the number of sequences within each haplotype. The number in brackets represent the number of mutations between different nodes or haplotypes.

When the *cox*1 and *cox*3 genes (1466 bp) were concatenated, 26 haplotypes among the 42 genotypes were identified (Fig. 1). The Albopictus Subgroup was composed of a mix of haplotypes of *Ae. albopictus* from Cambodia, Fiji, and Laos and two haplotypes of *Ae. unalom* (Fig. 1). The Scutellaris Subgroup was grouped in three distinct clusters. The first cluster contained two haplotypes of *Ae. futunae* from Wallis and Futuna*.* The second cluster was consisted of a combination of haplotypes of *Ae. polynesiensis* from Wallis and Futuna and *Ae. pseudoscutellaris* from Fiji. In this cluster, the distinction between *Ae. polynesiensis* from Wallis and Futuna and *Ae. pseudoscutellaris* from Fiji was ambiguous even when using the concatenated mitochondrial data (Fig. 1). The third cluster was composed of a mix of haplotypes of *Ae. malayensis* from Laos and *Ae. scutellaris* from New Caledonia.

### Estimating divergence times

**Figure 2:**
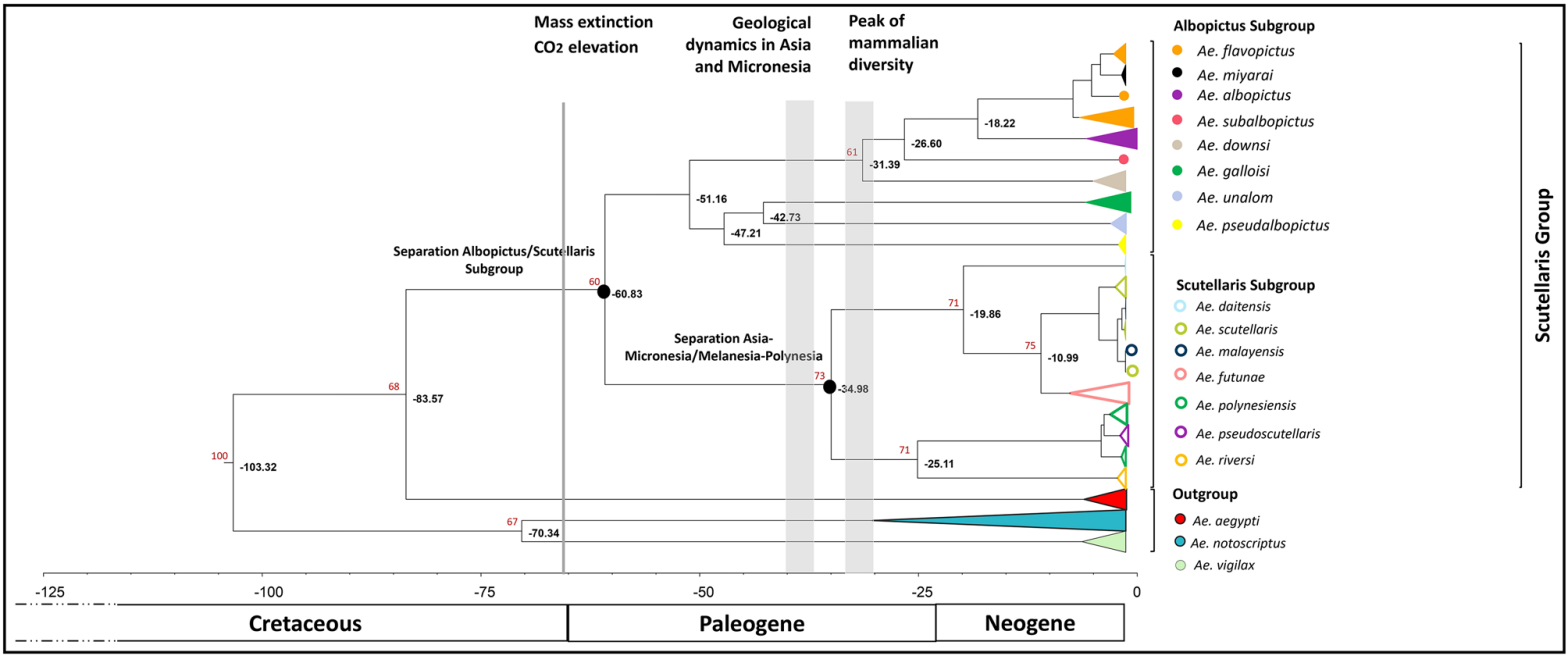
Evolutionary timescale of the Scutellaris Group using the *cox*1 gene. Tree derived from 1710 *cox*1 nucleotide sequences (379 bp) of the 5’*cox*1 gene and generated from BEAST program using the GTR + G + I evolutionary model. *Aedes aegypti, Ae. notoscriptus* and *Ae. vigilax* were used as outgroups. Two calibration points were used from prior Culicinae subfamily studies using mitochondrial full genomes: *Ae. polynesiensis-Ae. riversi*, and *Ae. notoscriptus-Ae. vigilax* speciation events^22^. For analysis, the percentage of replicate trees in which the associated taxa clustered together in the bootstrap test (1000 replicates) is shown for values over 60 (in red).

When examining the 5′ segment of the *cox*1 gene (379 bp after aligning our sequences with those from the GenBank database and trimming the extremity to have the same sequence length), the evolutionary timeline revealed that the divergence of the last common ancestor between *Ae. aegypti* (one of the species used as outgroup) and the *Aedes* of Scutellaris Group dated back to approximately 83 MYA (95%HPD: 61–108) (Fig. 2). The Albopictus and Scutellaris Subgroups were monophyletic, and speciation process between these two subgroups occurred around 61 MYA (95%HPD: 50–71).

The last common ancestor of the Albopictus Subgroup dated back approximately 51 MYA (95%HPD: 41–60). In this subgroup, the distinction between *Ae. flavopictus* and *Ae. miyarai* was ambiguous: we observed three distinct clusters of *Ae. flavopictus* mixed with one single cluster of *Ae. miyarai*.

Within the Scutellaris Subgroup, we identified two monophyletic clusters for which the last common ancestor dated back approximately 35 MYA (95%HPD: 32–35). The first cluster is mainly composed of species from Asia (*Ae. daitensis, Ae. malayensis, Ae. riversi*), except *Ae. scutellaris,* which was introduced to New Caledonia. The second cluster is exclusively composed of species from the Melanesia and Polynesia of the Pacific region (*Ae. futunae, Ae. polynesiensis* and *Ae. pseudoscutellaris*). The speciation of these two clusters took place nearly at the same period: around 20 MYA (95%HPD: 13–29) for the cluster comprising *Ae. daitensis, Ae. malayensis, Ae. riversi* and *Ae. scutellaris,* and approximately 25 MYA (95%HPD: 16–33) for the cluster composed of *Ae. futunae, Ae. polynesiensis* and *Ae. pseudoscutellaris* (Fig. 2)*.* The analysis of the 5’segment of the *cox*1 gene did not allow to distinguish *Ae. malayensis* and *Ae. scutellaris*: three clusters (containing a mix of the two species) were observed, with speciation occurring approximately 11 MYA (95%HPD: 7–14). Also, the analysis of this gene did not allow to distinguish *Ae. polynesiensis* and *Ae. pseudoscutellaris*, the last common ancestor of these two species dated back approximately 4 MYA (95%HPD: 1–5).

**Figure 3:**
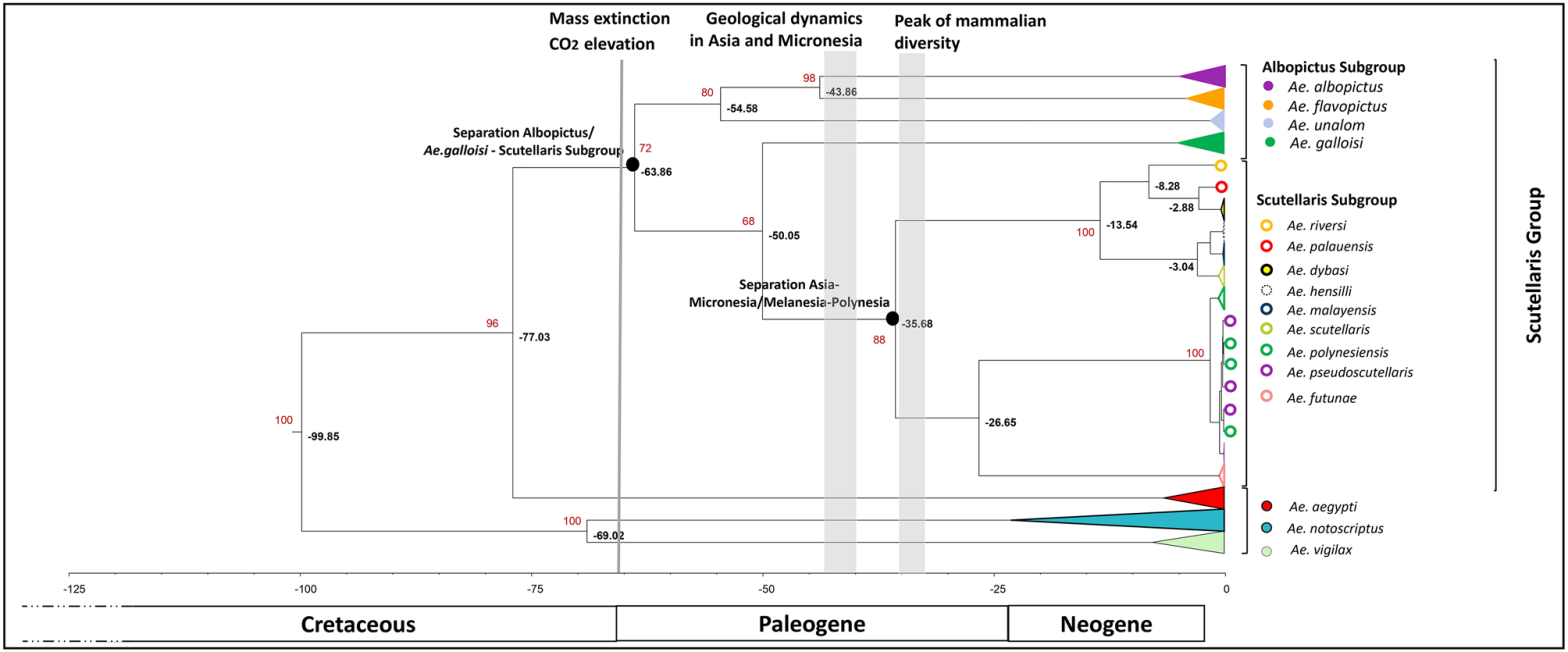
Evolutionary timescale of the Scutellaris using the *cox*1 gene. Tree derived from 167 *cox*1 nucleotide sequences (486 bp) of the 3′*cox*1 gene and generated from BEAST program using the GTR + G + I evolutionary model. *Aedes aegypti, Ae. notoscriptus* and *Ae. vigilax* were used as outgroups. Two calibration points were used from prior Culicinae subfamily studies using mitochondrial full genomes: *Ae. polynesiensis-Ae. riversi*, and *Ae. notoscriptus-Ae. vigilax* speciation events^22^. For analysis, the percentage of replicate trees in which the associated taxa clustered together in the bootstrap test (1000 replicates) is shown for values over 60 (in red).

The analysis of the 3′ part of the *cox*1 gene (486 bp after aligning our sequences with those from the GenBank database and trimming the extremity to have the same sequence length) revealed sequence differences compared to the 5′ fragment, influencing on the resulting phylogenetic tree structure. Contrary to the observation when analyzing the 5′ part of the *cox*1 gene, the Albopictus Subgroup formed a paraphyletic group, while the Scutellaris Subgroup was monophyletic (Fig. 3). The most recent common ancestor of *Ae. albopictus*–*Ae. flavopictus*–*Ae. unalom* of the Albopictus Subgroup and the species of Scutellaris Subgroup dated back around 64 MYA (95% HPD: 46–87). We also identified two monophyletic clusters within the Scutellaris Subgroup for which the last common ancestor dated back approximately 36 MYA (95%HPD: 32–50). The first cluster is mainly composed of a mix of species from Asia and Micronesia (*Ae dybasi, Ae. hensilli, Ae. malayensis, Ae. palauensis* and *Ae. riversi*), and *Ae. scutellaris* introduced in New Caledonia and their last common ancestor dated back approximately 13 MYA (95% HPD: 7–21). The second cluster included only species from Melanesia and Polynesia of the Pacific region (*Ae. futunae, Ae. polynesiensis* and *Ae. pseudoscutellaris*) for which the last common ancestor dated back around 27 MYA (95% HPD: 20–32). The distinction between *Ae. polynesiensis* and *Ae. pseudoscutellaris* was ambiguous.

## Discussion

This study explored the speciation patterns of the species within the Scutellaris Group by analyzing their mitochondrial genes. Our results showed that the speciation process of these species happened long before human migration. Human presence in the Asia–Pacific region began only 55–65 thousand years ago, with subsequent dispersal within the region^39^, whereas the radiation of Scutellaris species appeared from approximately 60 MYA. The significant temporal distinction between the evolutionary timeline of these species and human migration dismisses any correlation between human colonization from Asia to the Pacific region and the speciation of the Scutellaris Group, which was suggested in the 1960s^17^. Our research revealed a broader pattern of diversification for many other Scutellaris species, previously uninvestigated.

Due to the lack of complete genome data and limited availability of other mitochondrial genes in GenBank for most Scutellaris Group species, we used only *cox*1 gene fragments. This approach allowed us to incorporate existing sequences into our study. Despite using partial *cox*1 genes, our estimated dates closely align with the previously established timelines. Indeed, prior studies using various genetic markers, including mitochondrial DNA, nuclear DNA, and whole genome data, estimate the divergence time between *Ae. aegypti* and *Ae. albopictus* to be roughly between 81 and 67 MYA^22,23,40^. In our study, we observed a divergence between these two species at around 84 MYA and 77 MYA using the 5′*cox*1 and the 3′*cox*1 genes, respectively. Previous work has also identified the speciation between *Ae. albopictus* and *Ae. flavopictus* at between 32 and 28 MYA when using the concatenated mitochondrial and nuclear genes or the mitochondrial genes^23^. In our study, we observed a separation between these two species at approximately 18 MYA (95%HPD: 11–28) when analyzing the 5′*cox*1 or 44 MYA (95%HPD: 27–62) when using the 3′*cox*1 region. The consistency between our results and previous findings can confirm the reliability of our analyses.

We noted a variation in speciation dates, particularly within the Albopictus Subgroup, when comparing the 5′*cox1* gene (which has become the standard barcoding marker for insects^41^) and the 3′*cox*1 results. These variations could be attributed to the difference in nucleotide substitution patterns between these regions, previously highlighted in other insect families^42^, and also attributed to the different number of taxa and sequences included in the analyses. However, our results indicated overlapping dating intervals (95% HPD) for the two gene portions, particularly evident within the Scutellaris Subgroup, suggesting no significant difference between the dating results. For example, the speciation between *Ae. futunae* and the cluster *Ae. polynesiensis/Ae. pseudoscutellaris,* dated back to approximately 25 MYA (95%HPD: 16–33), when analyzing the 5′ *cox*1 and around 27 MYA (95%HPD: 20–33), when analyzing the 3′ *cox*1. Also, the speciation between *Ae. riversi* and the cluster *Ae. malayensis/Ae. scutellaris* was identified at approximately 11 MYA (95%HPD: 6–13) with the 5′ *cox*1 gene while the radiation between the cluster *Ae. riversi/Ae. palauensis/Ae. dybasi* and the cluster *Ae. hensilli/Ae. malayensis/Ae. scutellaris* was also estimated at around 13 MYA (95%HPD: 7–21) with the 3′*cox*1 gene.

Overall, our results demonstrated that most of the speciation occurred during the Paleogene and Neogene periods (66 MYA to 23 MYA). Previous works suggested that mammalian diversification began before the Cretaceous–Tertiary (K/T) boundary (before 66 MYA)^43,44^. The peak of mammalian diversification rate is estimated to have occurred between 33 MYA, to 30 MYA, and diversification rates remained high and constant until 8.55 MYA, before declining significantly^45^. A co-radiation between *Aedes* species of Scutellaris Group and mammals might, therefore, have occurred. However, further data and in-deep studies are necessary to provide enough evidence of this possible co-evolution.

Significant fluctuations in global climate and environmental perturbations due to an asteroid impact have been identified as a cause of mass extinctions, leading to the extinction of dinosaurs 66 MYA^46,47 ^. This asteroid impact was followed by extensive volcanic activity, known as the Deccan Traps, which influenced the atmospheric CO_2_ levels^48,49^. Elevated atmospheric CO_2_ levels have previously been associated with increased speciation in Culicidae^50^. Interestingly, our analysis identified a separation between the Albopictus and the Scutellaris Subgroup occurring just after this massive extinction event, estimated to have taken place around 64 MYA or 61 MYA.

In Eurasia and the Pacific region, the Paleogene and Neogene periods were characterized by geological dynamics that influenced the landscape^51–53^. One significant geological event was the creation of Palau's volcanic arc system in Micronesia. This happened about 40 MYA when the Pacific Plate moved under the Philippine Plate along the Kyushu-Palau Ridge^54^. At the same period, changes occurred along the Eurasian coastline, leading to the creation of the Philippine Plate and the Izu Arc, a volcanic chain in the western Pacific Ocean. This later event played a role in shaping Japan and forming the Japan Sea and Okhotsk Sea^55^. Within the Scutellaris Subgroup, we identified a separation after these periods. Indeed, a divergence between some species from Asia–Micronesia and Melanesia-Polynesia occurred 36 -35 MYA involving two clusters: the first cluster comprised *Ae. daitensis* and *Ae. riversi* (only known from Japan), *Ae. dybasi Ae. hensilli, Ae. palauensis* (present in Palau islands)^2,12,20^ and the second cluster consisted of *Ae. futunae* (endemic to Wallis and Futuna), *Ae. pseudoscutellaris* (only known from Fiji) and *Ae. polynesiensis* (widely distributed in the South Pacific)^12^.

Finally, we found low genetic divergence between *Ae. polynesiensis* and *Ae. pseudoscutellaris*, which corroborated with previous studies^19,21^. Even when using concatenated mitochondrial genes, our results demonstrated that the number of mutations observed between these two species was comparable to the mutations found among different *Ae. albopictus* populations. Researchers have hypothesized that these two species might either constitute a single species^18^ or are in the process of speciation^19^. To definitively determine the taxonomic status of these closely related species, which also exhibit morphological similarities^12^, a comprehensive genome sequencing approach would be essential. We, also, observed a difficulty in distinguishing *Ae. malayensis* and *Ae. scutellaris* in 5′*cox*1 region, when including in our analysis: *Ae. malayensis* sequences from Singapore^10^ and *Ae. scutellaris* from Thailand^56^. This ambiguity was not observed when using only the sequences generated from our laboratory: the phylogenetic tree generated from the 5′*cox*1 part was previously published in 2023^21^. Additionally, ambiguity in separating *Ae. flavopictus* and *Ae. miyarai* was also highlighted in our study. These findings could be attributed to the specimens being collected from different locations or the potential misidentification of the species due to their morphological similarities.

## Conclusion

Overall, we characterized the phylogeny of several *Aedes* species of the Scutellaris Group in the Asia–Pacific region. This research represents a significant step forward in understanding their evolution. Further studies with more extensive sampling and analyzing complete genome data should be done that will offer a more understanding of the evolutionary history of the Scutellaris Group.

### Supplementary Information

Supplementary Table 1.

Supplementary Table 2.

Supplementary Table 3.

Supplementary Table 4.

## Data Availability

Sequence data that support the findings of this study have been deposited in the GenBank database under the accession number PP355451 to PP355490 and PP357893 to PP357934. All the data are provided within the manuscript and the supplementary information.
